# Maxillary herpes zoster with nasociliary nerve involvement and *Klebsiella* superinfection: A rare occurrence

**DOI:** 10.1002/ccr3.9356

**Published:** 2024-09-25

**Authors:** Prosper Adjei, Stanley Anenyemele Asasu, Michael Lennart Ayeebo, Eunice Ansomah Donkor, Augustina Amoakohene‐Yeboah

**Affiliations:** ^1^ Department of Internal Medicine Methodist Hospital Wenchi Ghana; ^2^ Emergency Unit Methodist Hospital Wenchi Ghana

**Keywords:** herpes zoster, maxillary nerve, maxillary zoster, postherpetic neuralgia, trigeminal nerve, varicella‐zoster virus

## Abstract

Herpes zoster is an acute cutaneous viral disease resulting from reactivation of dormant varicella‐zoster virus. The maxillary nerve is the least frequently affected branch of the trigeminal nerve. Rarely, cutaneous lesions can be secondarily infected with *Klebsiella* species. This report discusses a case of maxillary zoster with nasociliary nerve involvement and *Klebsiella* superinfection.

## Graphical abstract

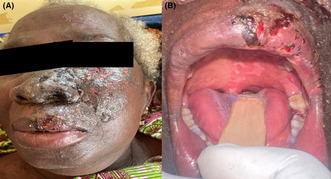

## 1 INTRODUCTION

Herpes zoster, also known as shingles, is an acute cutaneous viral disease which results from the reactivation of latent varicella‐zoster virus.^1^ In the younger healthy population, the incidence of herpes zoster ranges from 1.2 to 3.4 per 1000 person‐years. Among individuals older than 65 years, the incidence is 3.9 to 11.8 per 1000 person‐years.^2^

Following primary varicella infection, the varicella‐zoster virus establishes latency in the dorsal root or cranial nerve sensory ganglia. Failure of host immune defense mechanisms to suppress replication of the virus in the latent period results in the reactivation of varicella‐zoster virus, ultimately leading to the development of herpes zoster.^1^, ^2^, ^3^ In immunocompetent individuals, the lesions typically occur in a single dermatome, while in immunocompromised patients, they are usually multidermatomal.^4^ Herpes zoster predominantly affects the thoracic and cervical dermatomes in 45% and 23% of cases, respectively. Trigeminal nerve involvement occurs in 15% of cases.^4^ The ophthalmic nerve is the most commonly affected branch of the trigeminal nerve, while involvement of the maxillary division is very rare.^4^, ^5^ *Klebsiella* species are gram‐negative bacteria that rarely colonize the skin.^6^ Cutaneous infections caused by *Klebsiella* species are uncommon and often encountered in immunocompromised individuals.^7^, ^8^ We herein present a case of left maxillary herpes zoster with nasociliary nerve involvement and *Klebsiella* superinfection in an elderly patient who reported to the emergency unit of our hospital.

## 2 CASE HISTORY AND EXAMINATION

A previously healthy 79‐year‐old woman presented to the emergency unit of the Methodist Hospital with 3 days of worsening rash over the left mid‐facial region accompanied by severe pain in the affected area. She initially developed vesicles on the left half of the upper lip which subsequently worsened with the emergence of new vesicular lesions on the left side of the nose, ala nasi, cheek, lower eyelid and the tip of the nose. Additionally, she had multiple sores on the left side of the hard and soft palate. The rash was heralded by headache, low grade fever, malaise and burning pain over the left cheek. There was mild left ocular pain but no blurred vision, otalgia or vesicles on the ear. She denied any history of similar rash in the past. She did not have any malignancy and also was not on any immunosuppressive drugs. She sought medical care at a clinic where she was given oral flucloxacillin and topical acyclovir. She reported to our hospital for further evaluation when she noticed crusting of the skin eruptions.

**FIGURE 1:**
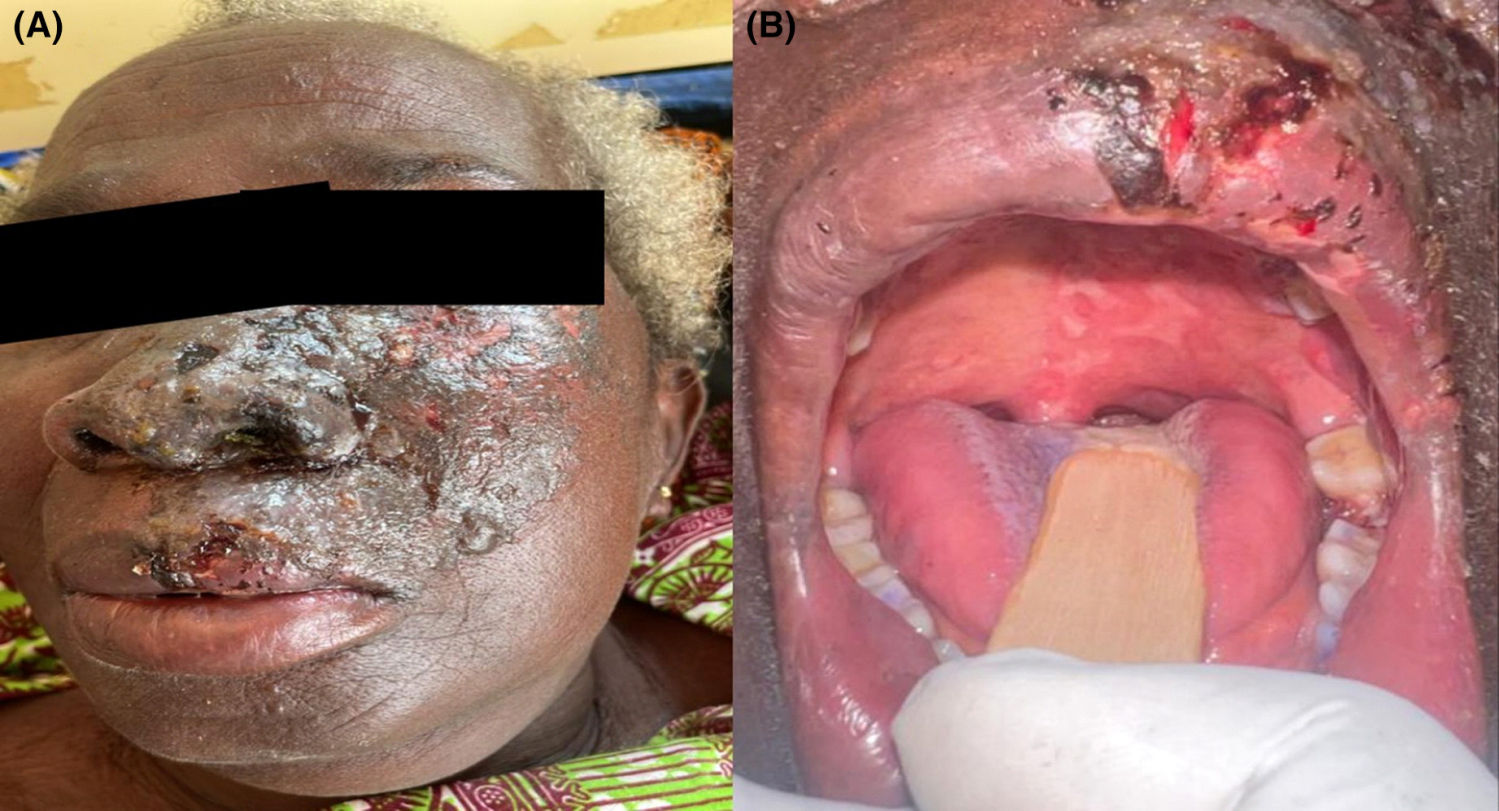
(A) Crusted lesions over the left middle‐third of the face along the distribution of the left maxillary branch of trigeminal nerve. (B) Ulcers limited to the left side of the hard and soft palate as well as left retromolar region and left half of the upper lip.

Skin examination showed mild edema of the left middle‐third of the face with unilateral crusted lesions over the left half of the upper lip, side of the nose, ala nasi, nasal vestibule, malar area, lower eyelid, and the tip of the nose (Figure 1A). Intra‐orally, there were multiple ulcers limited to the left side of the hard and soft palate without crossing the midline, as well as in the left retromolar region (Figure 1B). There was no facial asymmetry, left auricular tenderness or vesicles on the left pinna. Ophthalmic examination of the left eye revealed mild conjunctival congestion with normal cornea, uvea, and retina. Her vital signs were normal with unremarkable systemic examination.

## 3 INVESTIGATIONS, DIAGNOSIS, AND TREATMENT

Her fasting blood glucose and glycated hemoglobin were 6.3 mmol/L (reference range <7.0 mmol/L) and 3.75% (reference range <6.5%) respectively. Serological test for human immunodeficiency virus was negative. A swab culture of the lesions isolated *Klebsiella* species which was sensitive to levofloxacin, amikacin and ofloxacin. Complete blood count and serum electrolytes, as well as liver and renal biochemistries were normal. Standard 12‐lead electrocardiogram showed normal sinus rhythm with normal QT interval (QTc of 430 ms).

Given that the affected areas corresponded to the distribution of the left maxillary branch of the trigeminal nerve together with the involvement of the tip and side of the nose, a diagnosis of left maxillary herpes zoster with nasociliary nerve involvement and *Klebsiella* superinfection was made. She was treated with oral acyclovir 800 mg five times daily for 7 days, oral paracetamol 1 g three times daily for 5 days, oral levofloxacin 500 mg once daily for 7 days and 2% topical mupirocin eight hourly for 7 days.

## 4 OUTCOME AND FOLLOW‐UP

**FIGURE 2:**
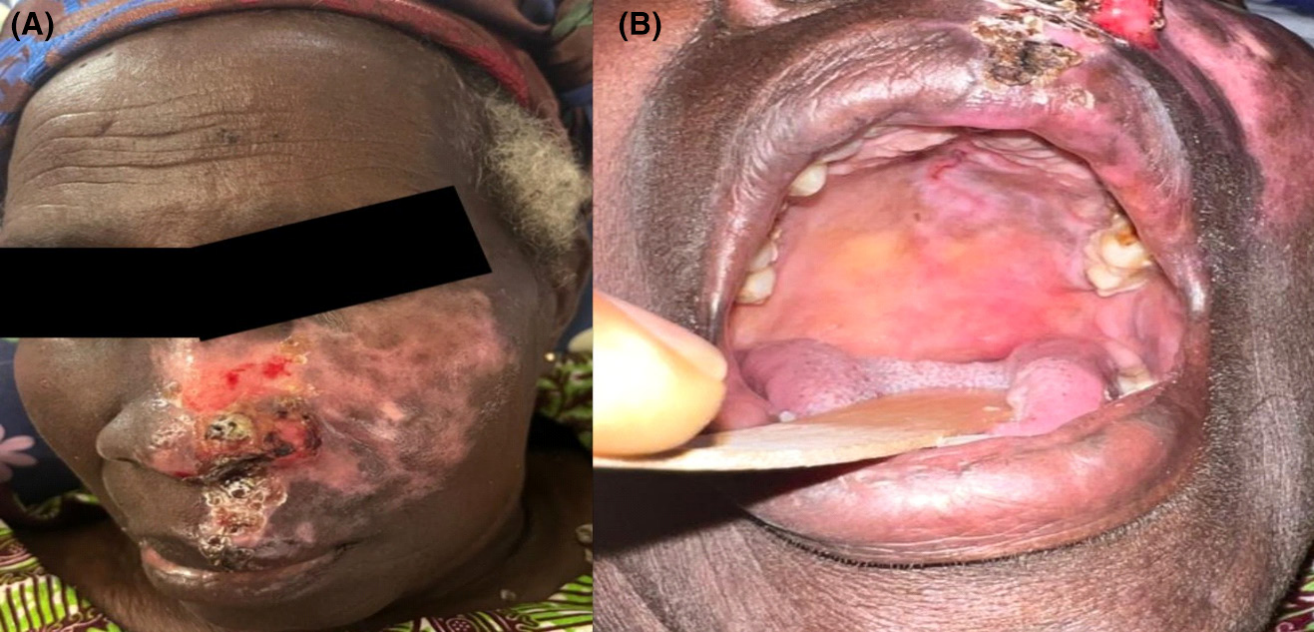
(A) Nearly complete healing of the left mid‐facial region. (B) Resolution of ulcers limited to the left side of the hard and soft palate as well as the retromolar region and left half of the upper lip.

**FIGURE 3:**
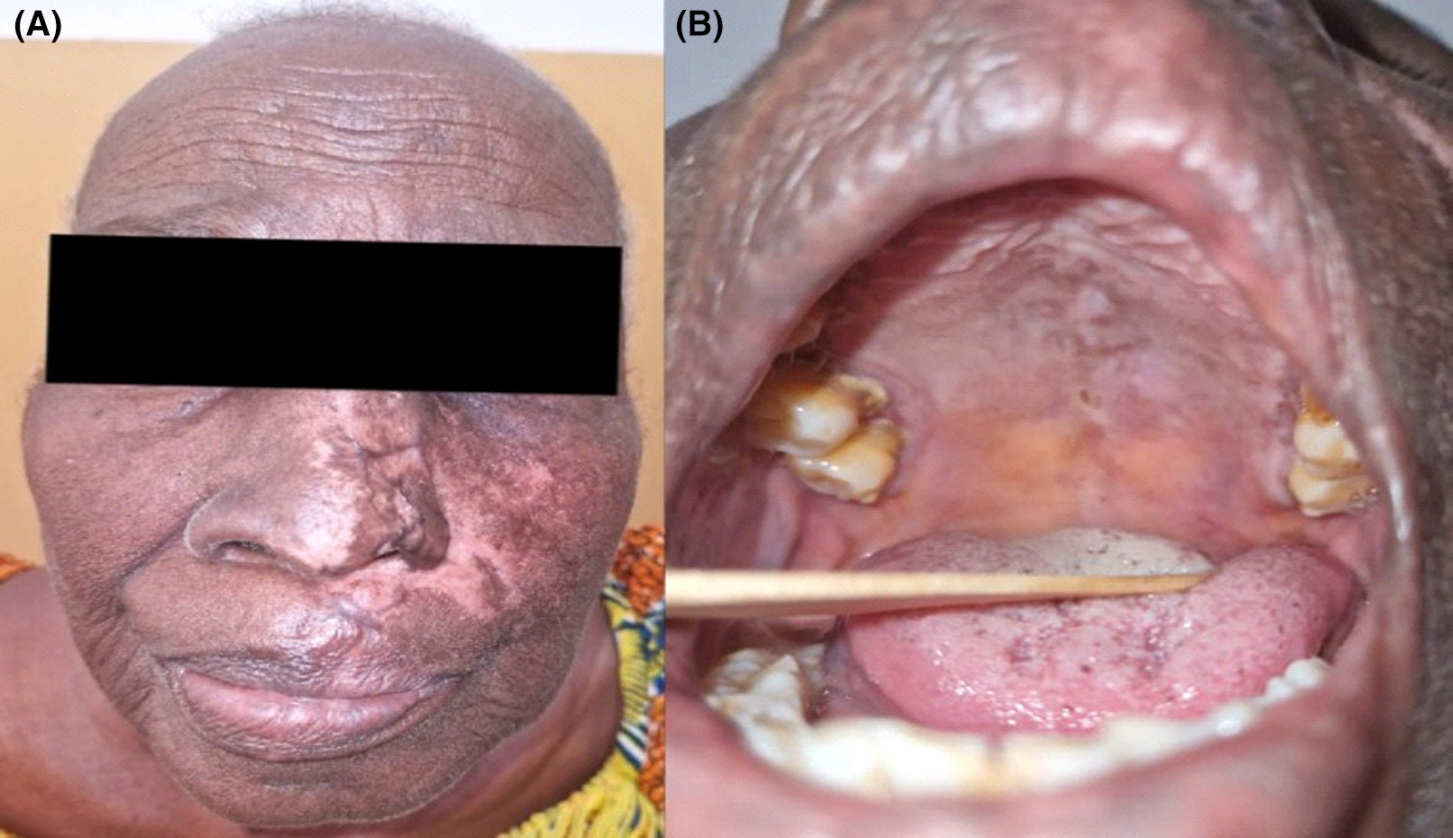
(A) Complete healing of left mid‐facial region with post‐inflammatory hypopigmentation at 8 weeks follow‐up. (B) Completely resolved oral ulcers.

At discharge after 10 days of hospitalization, there was resolution of the oral ulcers and conjunctival congestion with nearly complete healing of the left mid‐facial region (Figure 2A,B). Eight weeks after discharge, the skin over the left middle‐third of the face had completely healed with areas of post‐inflammatory hypopigmentation (Figure 3A). The oral ulcers had also completely resolved (Figure 3B). Additionally, she had developed postherpetic neuralgia (pain severity score of 7 out of 10) which greatly disrupted her sleep. This improved significantly (pain severity score reduced to 2 out of 10) after treatment with oral pregabalin 75 mg once daily for 2 months without any further interference with her sleep or daily activities.

## 5 DISCUSSION

Herpes zoster is a neurocutaneous disease caused by the reactivation of dormant varicella‐zoster virus.^1^, ^2^, ^3^, ^4^, ^5^ While decreased cell‐mediated immunity often leads to viral reactivation,^2^, ^9^ it may also occur spontaneously.^10^ Risk factors for herpes zoster include old age, human immunodeficiency virus infection, diabetes mellitus, malignancy, immunosuppressive therapy, and psychological stress.^1^, ^3^, ^5^, ^11^ The only risk factor that was apparent after clinical evaluation of our patient was her age (i.e., 79 years). Among the general population, the estimated lifetime risk of developing herpes zoster is 20%–30%. After 50 years of age, the risk significantly increases, approaching 50% at the age of 85 years.^12^

The clinical manifestations of herpes zoster are divided into three distinct phases namely preeruptive, acute eruptive and chronic phases.^1^, ^2^, ^5^ In the preeruptive phase, most patients may present with pain or burning sensation in the affected dermatome, fatigue, headache, low grade fever and myalgia as was observed in our patient. The acute eruptive phase is characterized by the appearance of erythematous macules and papules in a single, unilateral dermatome, with subsequent progression to vesicles, pustules, and crusts.^1^, ^2^, ^5^ Multidermatomal involvement is rare in immunocompetent individuals.^3^, ^13^ Herpes zoster affects the ophthalmic nerve about 20 times more often than the other two branches of the trigeminal nerve.^4^ Maxillary branch involvement is very rare and accounts for only 1.7% of cases.^14^ As noticed in our patient, the lesions in maxillary zoster are localized to the upper lip, cheek, side of nose, ala nasi, nasal vestibule, lower eyelid, cheek mucosa as well as on the hard and soft palate. The presence of lesions on the tip or side of the nose, known as Hutchinson's sign, indicates nasociliary nerve (a branch of ophthalmic division of trigeminal nerve) involvement. This confers a greater risk for ocular complications.^3^, ^14^ Although rare, corneal involvement may occur in maxillary zoster due to neuronal connections between nasal branches of maxillary nerve and external nasal branches of the anterior ethmoidal nerve (branch of nasociliary nerve)^5^, ^14^ In our case, the patient had only mild conjunctival congestion which resolved after treatment.

Generally, the diagnosis of herpes zoster is made clinically based on the history, characteristic morphology, and dermatomal distribution of the lesions. However, in individuals with atypical manifestations, laboratory confirmation via techniques such as polymerase chain reaction, direct fluorescent antibody testing, and viral culture is indicated.^1^, ^2^, ^5^ The therapeutic measures for herpes zoster include antiviral agents, analgesia for acute pain, and prevention of postherpetic neuralgia as well as bacterial superinfections.^5^, ^15^ Early initiation of antiviral agents within 72 h after the onset of symptoms has been shown to hasten the resolution of skin lesions and reduce the severity of zoster‐associated pain. Valacyclovir, acyclovir, famciclovir, and brivudine are the recommended antiviral agents for treating herpes zoster.^15^ As recommended in immunocompetent individuals, our patient received oral acyclovir 800 mg (five times daily) for a standard duration of 7 days,^15^ with complete resolution of the cutaneous lesions. In those with herpes zoster oticus (Ramsay Hunt syndrome) however, intravenous administration of acyclovir followed by oral treatment for 1–2 weeks has been shown to be effective.^15^

Postherpetic neuralgia is the commonest sequela of herpes zoster affecting about 50% of patients older than 60 years.^1^ Our patient's age was a major risk factor for developing postherpetic neuralgia as the risk increases 27‐fold in patients older than 50 years compared with those less than 50 years.^16^ *Staphylococcus aureus* and *Streptococcus pyogenes* are most frequently implicated in bacterial superinfections of herpes zoster.^17^ In the case of our patient, bacterial culture from the crusted skin lesions grew *Klebsiella* species which is an uncommon cause of skin infections.^7^, ^8^ Colonization of the skin by *Klebsiella* species is very rare and tends to be transient.^18^ The risk factors for *Klebsiella* infections include immunosuppression (e.g. corticosteroid therapy, organ transplant recipient), diabetes mellitus, alcoholism, prolonged antimicrobial therapy, prolonged use of medical devices like ventilators, intravenous catheters and history of hospitalization (especially to an intensive care unit).^18^, ^19^ The patient, however, did not have any of these factors. Her age most likely predisposed her to this rare bacterial skin infection because old age is associated with immunosenescence which leads to increased susceptibility to bacterial and viral infections.^20^

## 6 CONCLUSION

Herpes zoster involving the maxillary branch of the trigeminal nerve is very rare. Although *Klebsiella* species infrequently colonizes the skin, it can cause secondary infection of skin lesions in herpes zoster. Early diagnosis and prompt initiation of antiviral therapy reduces the duration of cutaneous symptoms as well as the severity of zoster‐associated pain.

## AUTHOR CONTRIBUTIONS

**Augustina Amoakohene‐Yeboah:** Data curation. **Prosper Adjei:** Conceptualization; investigation; writing – original draft; writing – review and editing. **Michael Lennart Ayeebo:** Data curation. **Stanley Anenyemele Asasu:** Data curation. **Eunice Ansomah Donkor:** Data curation.

## FUNDING INFORMATION

The authors received no financial support for the authorship and/or publication of this article.

## CONFLICT OF INTEREST STATEMENT

The authors declare no potential conflicts of interest with respect to the authorship and/or publication of this article.

## ETHICS STATEMENT

Our institution does not require ethical approval for reporting individual cases or case series.

## CONSENT

Written informed consent was obtained from the patient to publish this report in accordance with the journal's patient consent policy.

## Data Availability

Data sharing is not applicable.

## References

[1] Janniger CK , Eastern JS , Hospenthal DR , Moon JE . Herpes zoster. WebMD; 2021:2024 https://emedicine.medscape.com/article/1132465‐overview

[2] Nair PA , Patel BC . Herpes zoster. StatPearls Publishing; 2023 https://www.ncbi.nlm.nih.gov/books/NBK441824/ 28722854

[3] Pelloni LS , Pelloni R , Borradori L . Herpes zoster of the trigeminal nerve with multidermatomal involvement: a case report of an unusual presentation. BMC Dermatol. 2020;20:12. 33126864 10.1186/s12895-020-00110-1 PMC7602315

[4] Dube S , Pratyush R , Rajshekhar V . Multidermatomal herpes zoster ophthalmicus in an immunocompetent male. J Clin Ophthalmol Res. 2017;5:38‐40.

[5] Patro S , Jena PK , Misra GC , Rath KC , Khatua P . Herpes zoster infection involving the maxillary branch of the right trigeminal nerve ‐ a rare case report. Antiseptic. 2013;110:36‐38.

[6] Valenti M , Cortese A , Facheris P , et al. Atypical facial pustular folliculitis by *Klebsiella pneumoniae*: a case report. Dermatol Reports. 2023;16:9720. 38957644 10.4081/dr.2023.9720 PMC11216145

[7] Hu T , Wang M , Chen W , Zhao J , Xiong J . The clinical characteristic and outcome of skin and soft tissue infection in immunosuppressive patients with nephrotic syndrome. Clin Exp Nephrol. 2020;24:779‐788. 32342290 10.1007/s10157-020-01893-w

[8] Kim HS , Chang YJ , Chung CH . *Klebsiella pneumoniae* necrotizing fasciitis on the upper lip in a patient with uncontrolled diabetes. Arch Craniofac Surg. 2020;2:127‐131. 10.7181/acfs.2019.00696 PMC7206462 32380815

[9] Lovell B . Trigeminal herpes zoster: early recognition and treatment are crucial. BMJ Case Rep. 2015;2015:bcr2014208673. 10.1136/bcr-2014-208673 PMC4369019 25795749

[10] Kennedy PGE , Mogensen TH , Cohrs RJ . Recent issues in varicella‐zoster virus latency. Viruses. 2021;13:2018. 34696448 10.3390/v13102018 PMC8540691

[11] Papagianni M , Metallidis S , Tziomalos K . Herpes zoster and diabetes mellitus: a review. Diabetes Ther. 2018;9:545‐550. 29520743 10.1007/s13300-018-0394-4 PMC6104256

[12] John AR , Canaday DH . Herpes zoster in the older adult. Infect Dis Clin N Am. 2017;31:811‐826. 10.1016/j.idc.2017.07.016 PMC5724974 29079160

[13] Alhayyas M , Chaudhry M , Berdouk S . An atypical presentation of multidermatomal herpes zoster: a case report. Int J Emerg Med. 2020;13:58. 33256595 10.1186/s12245-020-00325-6 PMC7708088

[14] Dash S , Mohanty G . Corneal involvement in herpes zoster maxillaris: a rare occurrence. JCR. 2020;10:170‐172.

[15] Werner RN , Nikkels AF , Marinović B , et al. European consensus‐based (S2k) guideline on the management of herpes zoster ‐ guided by the European dermatology forum (EDF) in cooperation with the European academy of dermatology and venereology (EADV), part 2: treatment. J Eur Acad Dermatol Venereol. 2017;31:20‐29. 27579792 10.1111/jdv.13957

[16] Choo PW , Galil K , Donahue JG , Walker AM , Spiegelman D , Platt R . Risk factors for postherpetic neuralgia. Arch Intern Med. 1997;157:1217‐1224. 9183233

[17] Kalogeropoulos CD , Bassukas ID , Moschos MM , Tabbara KF . Eye and periocular skin involvement in herpes zoster infection. Med Hypothesis Discov Innov Ophthalmol. 2015;4:142‐156. 27800502 PMC5087099

[18] Chang D , Sharma L , Dela Cruz CS , Zhang D . Clinical epidemiology, risk factors and control strategies of *Klebsiella pneumoniae* infection. Front Microbiol. 2021;12:750662. 34992583 10.3389/fmicb.2021.750662 PMC8724557

[19] Centers for Disease Control and Prevention . Klebsiella , 2024, 2024. https://www.cdc.gov/klebsiella/about/index.html

[20] Lewis ED , Wu D , Meydani SN . Age‐associated alterations in immune function and inflammation. Prog Neuro‐Psychopharmacol Biol Psychiatry. 2022;118:110576. 10.1016/j.pnpbp.2022.110576 35588939

